# The Response of Duckweed *Lemna minor* to Microplastics and Its Potential Use as a Bioindicator of Microplastic Pollution

**DOI:** 10.3390/plants11212953

**Published:** 2022-11-02

**Authors:** Ula Rozman, Gabriela Kalčíková

**Affiliations:** Faculty of Chemistry and Chemical Technology, University of Ljubljana, 113 Večna pot, SI-1000 Ljubljana, Slovenia

**Keywords:** adhesion, aquatic, biomonitoring, microplastics, microbeads, phytoremediation

## Abstract

Biomonitoring has become an indispensable tool for detecting various environmental pollutants, but microplastics have been greatly neglected in this context. They are currently monitored using multistep physico-chemical methods that are time-consuming and expensive, making the search for new monitoring options of great interest. In this context, the aim of this study was to investigate the possibility of using an aquatic macrophyte as a bioindicator of microplastic pollution in freshwaters. Therefore, the effects and adhesion of three types of microplastics (polyethylene microbeads, tire wear particles, and polyethylene terephthalate fibers) and two types of natural particles (wood dust and cellulose particles) to duckweed *Lemna minor* were investigated. The results showed that fibers and natural particles had no effect on the specific growth rate, chlorophyll *a* content, and root length of duckweed, while a significant reduction in the latter was observed when duckweed was exposed to microbeads and tire wear particles. The percentage of adhered particles was ten times higher for polyethylene microbeads than for other microplastics and natural particles, suggesting that the adhesion of polyethylene microbeads to duckweed is specific. Because the majority of microplastics in freshwaters are made of polyethylene, the use of duckweed for their biomonitoring could provide important information on microplastic pollution in freshwaters.

## 1. Introduction

Plastic pollution has become one of the most important environmental issues of the last decades, with microplastics (MPs, pieces of plastic from 1 to 1000 µm [1]) being of increasing public and scientific concern due to their widespread occurrence [2]. They enter the environment through many pathways, including wastewaters [3], runoff [4], and atmospheric deposition [5]. MPs are also formed directly in the environment by the fragmentation of plastic items [6], but are degraded very slowly under natural conditions and therefore remain in aquatic ecosystems for a long time [7].

Traditionally, the presence and abundance of MPs in the aquatic ecosystem is monitored using physico-chemical methods [2]. However, such monitoring is challenging as MPs are not uniformly distributed in the water phase. Most of them float on the water surface because they are composed of low density polymers [8]. There, they interact with microorganisms that form a biofilm on their surface, resulting in increased size and density, and MPs can then sink deeper into the water body [9]. Further monitoring is even more difficult because MPs move dynamically from one environmental compartment to another, interact with biota, and become incorporated into sediment after settling [10,11]. However, reliable monitoring of MPs in the aquatic environment is crucial to identify the sources of MPs and to establish regulatory limits and measures to reduce them in the environment [12].

Recent research has shown that MPs interact with different organisms, so the use of biological indicators (bioindicators) could provide an alternative to traditional monitoring methods [13]. Ideally, the bioindicator should accumulate a high concentration of pollutant with low impact, be widespread and abundant, and be sessile to represent the local population [14]. Animal species such as fish and invertebrates may not meet the requirements for a bioindicator because the bioaccumulation of MPs may be low; many studies indicated that ingested MPs are also rapidly excreted [15,16]. On the other hand, recent studies showed that MPs can attach to biotic surfaces such as biofilms [17] or aquatic macrophytes [18]. The latter showed great potential to interact with MPs under laboratory conditions [19,20] and in field studies [11,21]. Macrophytes are minimally affected by environmentally relevant MP concentrations [22,23], and floating macrophytes are among the first organisms with which MPs interact when they enter the aquatic environment, as they collectively occupy the water surface. Therefore, floating macrophytes could be appropriate organisms for the biomonitoring of MPs in freshwaters.

In this context, the aim of this study was to investigate the potential use of the floating macrophyte duckweed *Lemna minor* as a bioindicator of MPs in freshwaters. Duckweed is widely used for toxicity testing [24] and as a bioindicator (e.g., for metal pollution [25]). It is tolerant to the presence of MPs [23], grows wild in European regions, and plays an important role as food for other organisms and habitat for various aquatic organisms [26]. For this purpose, the effect of various MPs and natural particles was tested to evaluate a specific response of duckweed to MPs exposure. The selected endpoints represent three main areas where MPs can affect plants: specific growth rate is an indicator of leaf damage, measurement of root length shows the effects on roots, and measurement of photosynthetic pigment content is a sensitive biomarker often used to detect adverse effects on photosynthesis [27,28]. Furthermore, the number of MPs and natural particles adhered to the duckweed biomass was monitored to evaluate the efficiency of duckweed in capturing MPs and thus its potential application for monitoring MP pollution. To our knowledge, this is the first study to consider floating aquatic macrophytes as potential bioindicators of MP pollution, and likely one of the first to monitor particulate matter in the aquatic environment.

## 2. Results

### 2.1. Characterization of Microplastics and Natural Particles

**Figure 1:**
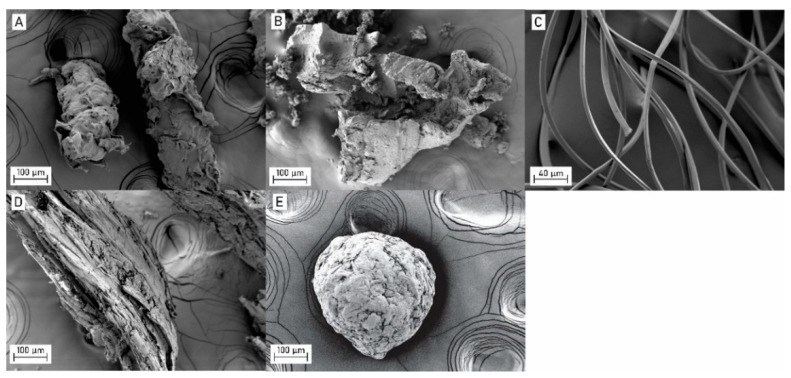
Microplastics and natural particles under the field-emission scanning electron microscope: (**A**) microbeads, (**B**) tire wear particles, (**C**) fibers, (**D**) wood dust, and (**E**) cellulose particles.

The characteristics of microplastics (MPs) and natural particles are shown in Figure 1 and Table 1. Microbeads, tire wear particles, and wood dust had irregular shapes, while fibers were smooth and uniform. Cellulose particles had the shape of beads, but with irregularities on the surface (Figure 1).

Microbeads, wood dust, and cellulose particles had a similar number of particles per mass, while fibers and tire wear particles had a higher number of particles per mass (Table 1). The mean size of MPs and natural particles was different, ranging from 47 ± 22 µm to 296 ± 45 µm, while for fibers the mean length and diameter were determined separately due to large length-to-diameter ratio (Table 1). The chemical characterization of MPs was previously performed by Rozman et al. and, based on the results of FTIR analysis, the microbeads and fibers were pure low-density polyethylene and polyethylene terephthalate, respectively, while the FTIR spectrum of tire wear particles confirmed that particles were derived from rubber tires [23].

### 2.2. Effects of Microplastics and Natural Particles on Duckweed

All particles introduced into the test floated on the water surface and were thus in contact with duckweed. After exposure to MPs and natural particles, the specific growth of duckweed *Lemna minor* was not significantly affected (Table 2). The average specific growth rates in the control, microbeads, tire wear particles, fibers, wood dust, and cellulose particles treatments were 0.324 ± 0.008 day^−1^, 0.313 ± 0.015 day^−1^, 0.302 ± 0.022 day^−1^, 0.302 ± 0.017 day^−1^, 0.310 ± 0.026 day^−1^, and 0.306 ± 0.033 day^−1^, respectively. No significant reduction in chlorophyll *a* content was observed in all treatments compared to the control treatment. The chlorophyll *a* content was 0.503 ± 0.027 mg/g, 0.518 ± 0.027 mg/g, 0.535 ± 0.031 mg/g, 0.450 ± 0.038 mg/g, 0.505 ± 0.063 mg/g, and 0.581 ± 0.185 mg/g in the control, microbeads, tire wear particles, fibers, wood dust, and cellulose particles treatments, respectively. On the other hand, microbeads (DF = 6, *p* = 0.00868) and tire wear particles (DF = 6, *p* = 0.000039) caused a significant reduction in the length of duckweed roots (mean root length of microbeads and tire wear particles were 23.3 ± 2.6 mm and 21.8 ± 2.0 mm, respectively). The effects of fibers on the roots of duckweed were comparable to those of natural particles, as the mean root length in the control, fibers, wood dust, and cellulose particles treatments was 29.2 ± 1.6 mm, 27.6 ± 0.6 mm, 28.2 ± 1.1 mm, and 29.3 ± 3.3 mm, respectively.

### 2.3. Adhesion of Particles to Duckweed

**Figure 2:**
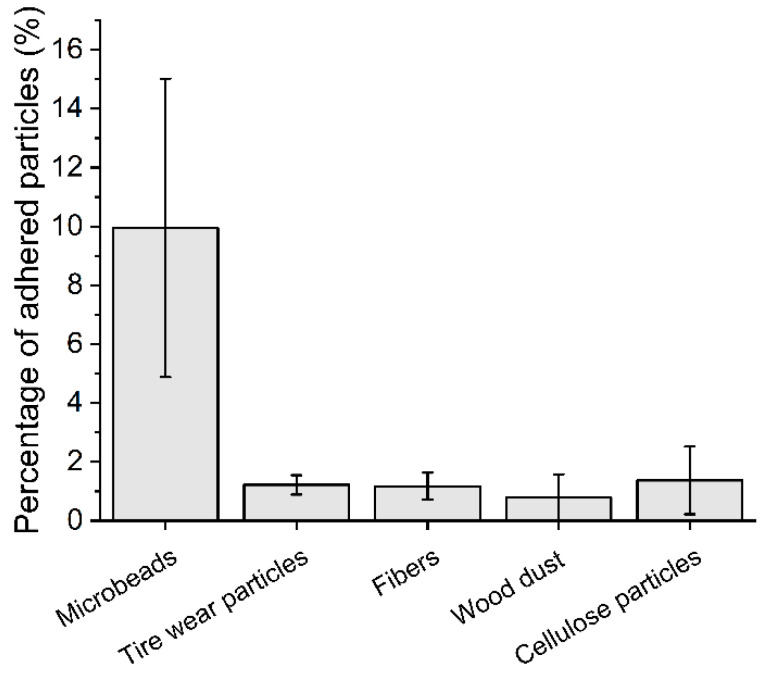
The percentage of adhered microplastics and natural particles to duckweed.

**Figure 3:**
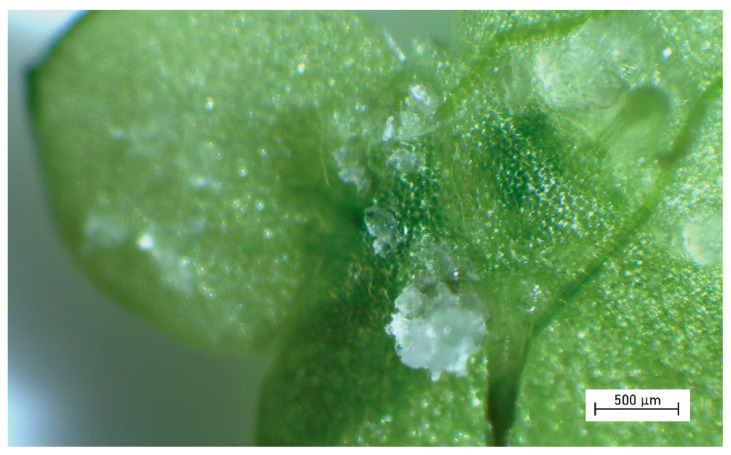
Microbeads adhered to fronds of duckweed.

The percentage of adhered particles to duckweed is shown in Figure 2. The adhesion of tire wear particles, fibers, wood dust, and cellulose particles was similar (approximately 1%); however, the percentage of adhered microbeads was 10-times higher. Due to the high number of adhered microbeads (Figure 3), the effect of gentle shaking was further investigated, and the number of adhered microbeads with shaking was slightly higher than the number of adhered microbeads without shaking (13.0 ± 3.5% and 10.0 ± 5.1%, respectively); however, it was not statistically significant (U = 11.5, *p* = 0.3350).

## 3. Discussion

Large quantities of MPs continuously enter aquatic ecosystems, but their monitoring is difficult, as MP concentrations are highly heterogeneous [2], vary over time [29], and fluctuate even within a site [30]. Therefore, the use of biomonitoring could be beneficial because organisms live at the site for a long period of time and are thus in long-term contact with MPs. The use of organisms as bioindicators of MPs is still in its early stages, with a focus on the marine environment and the use of animal species [31,32]. Therefore, in this study, we focused for the first time on the use of the floating macrophyte duckweed *Lemna minor* as a potential bioindicator of MP pollution in freshwaters.

In the first part of this study, the specific response of duckweed to the presence of MPs was studied, because it is possible to monitor pollutants based on the effect they trigger (e.g., use of biomarkers) [33]. Results showed that MPs did not elicit a negative response on duckweed specific growth rate, including chlorophyll *a* content. Microbeads and tire wear particles caused a reduction in root length, while fibers and natural particles did not. In general, MPs can induce an adverse response by mechanical and/or chemical stress. The latter can occur through the leaching of additives [34], but this was not the case here because all MPs were previously tested for possible leaching and their leachates had no effect on duckweed [23]. It is very unlikely that the MPs used in this study penetrated the roots because they are too large to pass through the cell wall. This is in agreement with the results of Dovidat et al. [34], who investigated the uptake of micro- and nano-plastics by confocal microscopy in *Spirodela polyrhiza* and found that plastic particles adhered only externally, while no micro- or even nano-plastics were allocated inside the roots. Therefore, it is plausible that root length was affected due to mechanical stress (external abrasion of roots), and the effect may be related to the surface morphology of the MPs, as particles with sharp edges could affect root length and root cell viability [27]; microbeads and tire wear particles had sharp edges, while fibers were perfectly smooth. Although wood particles also had sharp edges, they did not affect duckweed, most probably due to the softness of wood [35]. In previous studies, long-term exposure of duckweed to microbeads also showed no effects on carbohydrates, lipids, proteins, the activity of electron transport system, and antioxidant capacity [20], and thus the most sensitive endpoint following exposure of duckweed to MPs appear to be the root length. However, in the aquatic ecosystem, MPs are inhabited by microorganisms that can form a biofilm [36] that covers their surface and mitigates the effect of sharp MPs [37]. Therefore, the MP-induced reduction in duckweed root length may not occur in the environment, making monitoring of this endpoint in duckweed impractical for MP biomonitoring.

Furthermore, we focused on the monitoring of MPs adhered to duckweed biomass as previous studies have shown that MPs can interact with organisms via bio-adhesion [38,39]. The results showed that only microbeads (made of polyethylene) were adhered to a greater extent and that the percentage of adhered tire wear particles, fibers, wood dust, and cellulose particles was comparable. It is plausible that the initial interactions between MPs and duckweed are electrostatic in nature, as negatively charged plant biomass attracts positively charged MPs [40]. This would also explain the low adhesion of natural particles made of wood and cellulose as they carry the same charge as plant biomass ([41] and [42], respectively). Similarly, used rubber tires had a negative charge as they obtain an excess of electrons from the road [43], while polyethylene microbeads can be positively charged [44] (no data on the charge of PET fibers were found). The interaction between microbeads and duckweed can be rather strong as water movement did not affect the percentage of adhered microbeads, and thus natural water flow may not have a significant impact on MPs adhesion.

From the results, it is apparent that polyethylene microbeads are the only particles that adhered to plant biomass to a higher extend. These microbeads originated from a cosmetic product but they are also similar to the MPs sampled in freshwaters; they had the shape of irregular fragments and a similar particle size (~150 µm) to MPs detected in rivers and lakes [45,46], and the concentration used here (100 mg/L = 6 800 MPs/L, calculated according to Table 1) can also be considered relevant to MP hotspots, as Pivokonsky et al. detected up to 3 605 MPs/L in freshwater lakes [47]. Thus, the environmental relevance of this study is undisputed; however, it should be noted that many physico-chemical and biological processes occur in the environment and that further research, including initial field studies, is needed for the successful use of duckweeds as bioindicators of MP pollution. Currently, we can support our conclusion only with the results of monitoring studies in the marine environment, where the strong interactions between MPs and plant biomass were also confirmed. For example, Goss et al. monitored MPs adhered to seagrass *Thalassia testudinum* and found a number of MPs attached to the blades and overgrown by periphyton [18]. Huang et al. monitored the abundance and diversity of MPs in seagrass *Enhalus acodoides* and found that vegetated sites had up to 2.9 times more MPs than bare sites, with polyethylene MPs being the most abundant MP type.

The use of duckweed as a bioindicator for polyethylene MPs seems promising, not only because of the intensive adhesion to plant biomass, but also because polyethylene MPs float on the water surface and can be immediately captured by duckweed before they begin to sink or are transported further. Therefore, duckweed-derived MPs can indicate relatively fresh/immediate MP contamination, and the biomonitoring could identify nearby sources of MPs. In addition to the use for biomonitoring, the use of duckweed to collect MPs could also be used for phytoremediation. Removal of contaminated biomass could reduce the number of MPs by preventing them from spreading further into aquatic ecosystems where their removal is currently impossible.

## 4. Materials and Methods

### 4.1. Microplastics and Natural Particles

Three different types of microplastics (MPs) and two types of natural particles were used in this study. Microbeads and cellulose particles were extracted from two different facial scrubs where they serve as abrasives. Both were extracted using the same methods described in Kalčíková et al. [27]. Briefly, 50 mL of a facial scrub was dissolved in a warm deionized water under a stirring condition (400 rpm). The solution was filtered through filter paper (pore size 12–25 µm, Macherey-Nagel, Düren, Germany), and both types of particles were washed three times with deionized water and dried at 40 ± 2 °C overnight. Tire wear particles were obtained from a local car repair service, and particles were prepared by cutting pieces of old used tires. Particles were separated by size using sieves with mesh sizes of 355 and 125 µm, and the middle fraction (between 125 and 355 µm) was further used in this study. Fibers were obtained from synthetic clothing by milling at 8000 rpm for 6 min (Tube Mill 100 control, IKA, Staufen, Germany) [23]. The wood dust was from beech (*Fagus* sp.), and the particles were prepared by drilling holes in a beech wood slab, and the resulting sawdust was sieved (800 µm) to obtain a smaller size fraction of the dust.

MPs and natural particles were characterized in terms of their size, number of particles per mass, shape, morphology, and chemical composition, as described in Rozman et al. [23]. Size was determined by a laser diffraction analyzer (S3500 Bluewave, Microtrac, Haan/Duesseldorf, Germany) using a dry unit. The measurement was repeated three times, and the results were expressed as the number of particle size distribution. To determine the number of particles per mass, an amount of 1–2 mg of particles was weighed and counted using a stereo microscope (SMZ-171, Motic, Xiamen, China). The procedure was repeated ten-times to include at least 1000 particles in the analysis. The number of particles per mass of fibers was determined by measuring the length and diameter of numerous fibers under an optical microscope (Imager.Z2m, Zeiss, Oberkochen, Germany). The mass of each particle was calculated based on their density [48] and the volume of the fiber, resulting in the calculation of the mean number of particles per mg. The shape and morphology of the particles were examined using a field-emission scanning electron microscope (FE-SEM, Ultra plus, Zeiss, Oberkochen, Germany) at an accelerating voltage of 2 kV using a secondary detector. Before analysis, MPs and natural particles were coated with a thin Au/Pb layer. Chemical composition was determined by a Fourier transform infrared (FTIR) spectrometer (Spectrum Two FT-IR, PerkinElmer, Beaconsfield, UK) in the wavelength range from 4000 cm^−1^ to 400 cm^−1^ (resolution 2 cm^−1^, 10 scans). The background and ATR correlation of the spectra was performed [23].

### 4.2. Duckweed Lemna minor

The duckweed *Lemna minor* used in this study originated from a permanent laboratory culture. Plants were grown in 2-litre rectangular vessels in Steinberg medium [49] at 24 ± 1 °C and a 16/8-h photoperiod (light/dark) with a light intensity of 3500 ± 500 lx. The medium was changed weekly, and overgrown biomass was removed, leaving at least 2/3 of the surface free for further growth.

### 4.3. Ecotoxicity Test

The ecotoxicity test largely followed the OECD Guidelines No. 221 [49], with some minor modification. The concentration of 100 mg/L of MPs and natural particles was used for the experiment as it is recommended as a limit concentration by the OECD [50] and used in other MPs studies [51,52]. MPs and natural particles were directly weighted into 100 mL glass beakers and, afterwards, 50 mL of Steinberg medium was added into each glass beaker. In all treatments, the roots of duckweed were removed before exposure, and randomly selected plants with a total of ten fronds were placed into each beaker. They were incubated at the same temperature and photoperiod as in the permanent laboratory culture with a high light intensity of 7000 ± 500 lx and humidity of >70% to minimize evaporation of the medium from the test vessels. Each treatment was replicated four times. After seven days of incubation, the number of fronds was counted and the specific growth rate was calculated based on the OECD Guidelines No. 221 [49]. The root length of ten randomly selected plants in each test vessel was measured using millimeter paper [35]. To determine chlorophyll *a* content, approximately 15 mg of the fresh plant was homogenized in cold 95% *(v*/*v)* ethanol. After 24 h incubation in a freezer at −18 ± 2 °C, the absorbance of the supernatant was measured at 664.2 nm and 648.6 nm using a spectrophotometer (Cary 50 UV-Vis spectrophotometer, Agilent Technologies, Santa Clara, CA, USA) [53], and chlorophyll *a* content (mg/g) was calculated according to Lichtenthaler [54].

### 4.4. Adhesion of Particles to Duckweed

Based on our preliminary experiments, the maximum number of adhered polyethylene MPs to duckweed was reached after 24 h (not yet published); therefore, incubation time for adhesion experiments was the same. The experiment was set up in a similar way to the ecotoxicity test: the concentration of MPs or natural particles was 100 mg/L, the test was performed in test vessels containing 50 mL of Steinberg medium, and ten fronds were added to each test vessel, but in this case the roots were not removed. The test vessels were incubated for 24 h under the same conditions as in the ecotoxicity test (16/8 h, 7000 ± 500 lx, 24 ± 1 °C, humidity of >70%). After incubation, the number of adhered MPs and natural particles was determined. Plant biomass from each test vessel was washed with deionized water, and the washing water was filtered (S-Pak filter, pore size 0.22 µm, Merk Millipore, Burlington, MA, USA). The filters with retained particles were dried at room temperature for 24 h, and the particles were counted using the stereo microscope. The plant biomass was then weighed and digested by Fenton oxidation, as described in Rozman et al. [20]. Briefly, 2 mL of 0.015 g/mL Fe_2_SO_4_ · 7H_2_O (with 3 mL/L of H_2_SO_4_ (97% *v*/*v*)) and 2 mL of 30% (*w*/*w*) H_2_O_2_ [55] were added to each test tube containing a previously weighed plant. The digestion process lasted 24 h at room temperature (22 ± 2 °C). The digestate was filtered (S-Pak filter, pore size 0.22 µm, Merk Millipore, Burlington, MA, USA), the filters were dried at room temperature for 24 h, and the number of particles on the filter paper was counted under the stereo microscope. The total number of adhered particles to duckweed was the sum of the washed particles and the particles remaining in the digestate. Each treatment was replicated four times, and the results were expressed as the number of particles per fresh weight of duckweed [20].

Due to the extensive adhesion of polyethylene microbeads to duckweed, an additional experiment was conducted to investigate the effects of water movement. The experiment was performed under the same conditions as described above, except that it was slightly shaken (70 rpm) on an orbital shaker (Orbit 19000, Labnet, Edison, NJ, USA).

The plant biomass in the control treatment was processed in the same way to monitor for possible airborne contamination, but no particles were detected. MPs and natural particles only (excluding plant tissue) were also subjected to Fenton oxidation under the same conditions as described above (each replicated five times) to evaluate the effects of the process (filtration, digestion, etc.) on particle loss. The mass was reduced by 2.8 ± 1.7%, 6.8 ± 1.2%, 3.6 ± 3.7%, 7.5 ± 2.7%, and 2.3 ± 1.4% for microbeads, tire wear particles, fibers, wood dust, and cellulose particles, respectively, so the effect of the procedure was considered to be of minor importance.

The percentage of MPs or natural particles adhered to plant biomass was calculated by comparing the total number of MPs or natural particles in the test vessel (calculated based on the number of particles per mass, Table 1) and their number adhered to plant biomass:

*P* = *(n*_0_ − *n_x_*)/*n*_0_ ·100 (1)

where *P* (%) is the percentage of adhered MPs or natural particles to plant biomass, *n_0_* (/) is the number of MPs or natural particles introduced into the vessels, and *n_x_* (/) is the number of MPs or natural particles adhered to plant biomass [20].

### 4.5. Data Analysis

To analyze statistically significant differences between treated and control groups when testing the effects of MPs and natural particles on duckweed, normality was tested using the Shapiro–Wilk test and homogeneity of variances with Levene’s test. When normality and/or homogeneity of variances were not achieved, statistical differences compared to control were tested with the Mann–Whitney *U* test, but when the data were considered as normal and homogeneous, Student’s *t*-test was used. Differences were considered statistically significant if *p* < 0.05. All data analysis was preformed using OriginPro 2021b software (OriginLab Corp., Northampton, MA, USA).

## 5. Conclusions

The biomonitoring of various environmental pollutants is a widely used strategy, and bioindicators provide important information about the quality of the surrounding environment. So far, bioindicators have been used to monitor organic and inorganic substances, but particulate matter has remained unnoticed, and thus the biomonitoring of MPs is also in its infancy. This study presents a proof of concept for the monitoring of MPs in freshwaters by using duckweed *Lemna minor*. The results showed that polyethylene microbeads adhere to the plant biomass to a significant extent compared to other MPs or natural particles. Because MPs can change their properties and disperse in the water column over time, it is plausible to consider duckweed as a bioindicator of fresh MP pollution when MPs are still associated with the water-air interface and can be in close contact with the floating macrophyte. Because biomonitoring and the use of bioindicators are an important way to monitor pollutants as complex as MPs, both a systematic survey and a field study under environmentally relevant conditions are recommended.

## Figures and Tables

**Table 1:** Characteristics of microplastics (microbeads, tire wear particles, fibers) and natural particles (wood dust, cellulose particles).

	Size (Mean ± SD) (µm)	Number of Particles per Mass (particles/mg)	Chemical Composition
Microbeads	149 ± 75	68	Low density Polyethylene (PE)
Tire wear particles	47 ± 22	445	Rubber
Fibers	Length: 5362 ± 1082 Diameter: 9.6 ± 3.5	581	Polyethylene Terephthalate (PET)
Wood dust	253 ± 142	44	/
Cellulose particles	296 ± 45	48	/

/—not determined.

**Table 2:** Inhibition of specific growth rate, root length, and chlorophyll *a* content after exposure to microplastics (microbeads, tire wear particles, fibers) and natural particles (wood dust, cellulose particles).

	Inhibition (%)		
	Specific Growth Rate	Root Length	Chlorophyll *a*
Microbeads	3.4	20.2 *	0
Tire wear particles	6.8	25.3 *	0
Fibers	6.8	5.5	10.5
Wood dust	4.3	3.4	0
Cellulose particles	5.6	0	0

* Statistical significance compared to control (*p* < 0.05).

## Data Availability

The data presented in this study are available on request from the corresponding author.
